# P-620. High Risk, Low Compliance: Surprising Post-Pandemic Influenza Vaccine Trends in a Children's Hospital

**DOI:** 10.1093/ofid/ofae631.818

**Published:** 2025-01-29

**Authors:** Brianna Leyden, Wendi Gornick, Martin T Tran, Jasjit Singh

**Affiliations:** Children's Hospital of Orange County, ORANGE, California; Children's Hospital Orange County, Orange, California; CHOC Children's Hospital, Orange, California; CHOC Children's Hospital, Orange, California

## Abstract

**Background:**

Post-pandemic, the CDC reports a decline in influenza vaccinations in children 6 months to 17 years by 6.3% from 2019-2020 to 2022-2023. We describe concerning widespread declines in our institution in inpatient influenza vaccinations, outpatient oncology influenza vaccine rates in those receiving active chemotherapy, and employee vaccinations. We present these trends in the context of local influenza epidemiology, influenza admissions, and reasons for refusal.

**Figure:**
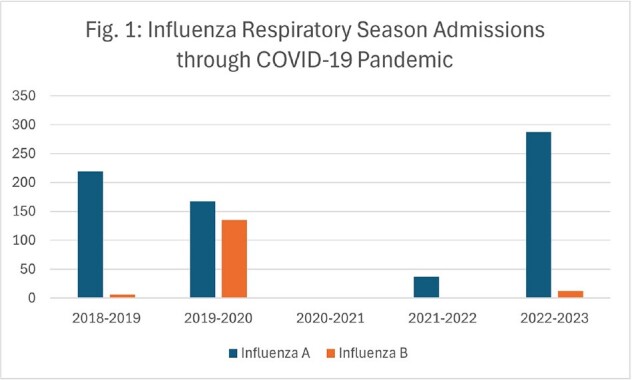

**Methods:**

Our institution, a free-standing children’s hospital, collected inpatient influenza vaccinations based on calendar years 2019-2023 and influenza admission rates over winter seasons 2018-2019 to 2022-2023. Additionally, employee vaccination rates, including reasons for refusal, were collected fall/winter 2018-2019 to 2023-2024. Lastly, oncology clinic influenza vaccination rates among the high-risk patients receiving active chemotherapy were collected from Sept-Dec 31 2020-2023.

**Figure:**
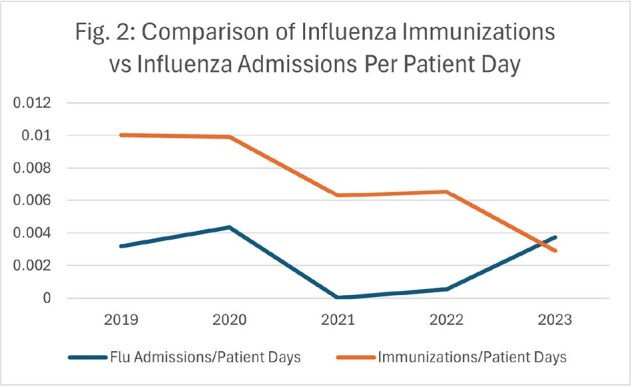

**Results:**

Although influenza admissions plummeted from 302 in winter 2019-2020 to 0 in winter 2020-2021, these admissions have since rebounded (Fig. 1). While inpatient influenza immunizations have continued to decline to a low of 0.0029 vaccines/patient day, they are now outpaced by influenza admissions at 0.0037 admissions/patient day (Fig. 2). This trend in decreased vaccines is echoed in oncologic patients on active chemotherapy, with non-medical refusals increasing from 5% in 2020 to 16% in 2023, and in employees, with refusals increasing from 1.9% in 2019 to 5.3% in 2024 (Fig. 3). Although employee rates for medical exemption are consistent, non-medical reasons for refusals have increased 6-fold from 2019 to 2024 (Fig. 4).

**Figure:**
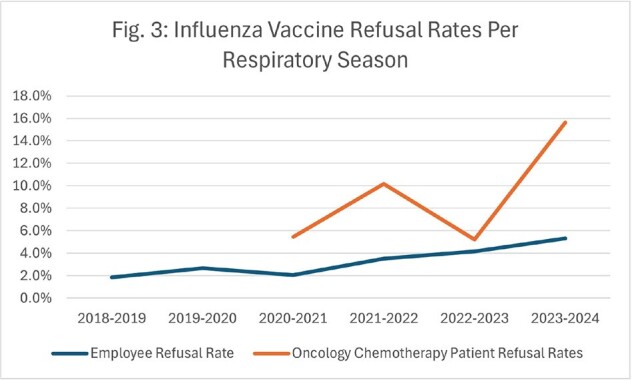

**Conclusion:**

We saw a decline in influenza vaccine rates for hospitalized patients, immunocompromised outpatient oncology patients on active chemotherapy, and employees, despite increased influenza burden in the community post-pandemic. The latter were almost exclusively due to non-medical reasons for refusal. Further study needs to be done to elucidate post-pandemic vaccine trends and identify missed opportunities for immunization, particularly in high-risk patients and their contacts, with emphasis on bolstering influenza vaccine confidence in not only patients, but staff.

**Figure:**
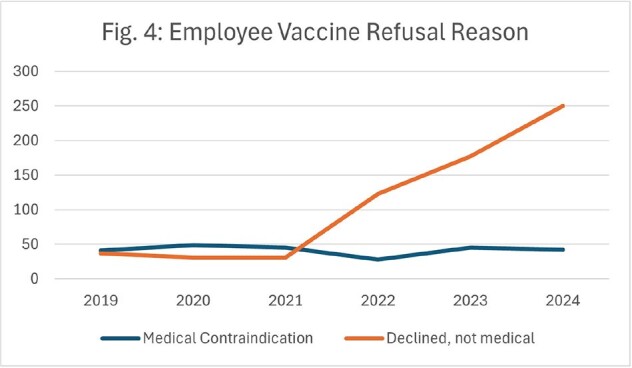

**Disclosures:**

**All Authors**: No reported disclosures

